# CSC software corrects off-target mediated gRNA depletion in CRISPR-Cas9 essentiality screens

**DOI:** 10.1038/s41467-021-26722-w

**Published:** 2021-11-09

**Authors:** Alexendar R. Perez, Laura Sala, Richard K. Perez, Joana A. Vidigal

**Affiliations:** 1 Laboratory of Biochemistry and Molecular Biology, National Cancer Institute, Bethesda, MD USA; 2 Department of Anesthesia and Perioperative Care, University of California, San Francisco, San Francisco, CA USA; 3 School of Medicine, University of California, San Francisco, San Francisco, CA USA; 4 Department of Anesthesiology, Perioperative and Pain Medicine, Stanford University, Palo Alto, CA USA

**Keywords:** Functional genomics, CRISPR-Cas systems

## Abstract

Off-target effects are well established confounders of CRISPR negative selection screens that impair the identification of essential genomic loci. In particular, non-coding regulatory elements and repetitive regions are often difficult to target with specific gRNAs, effectively precluding the unbiased screening of a large portion of the genome. To address this, we developed CRISPR Specificity Correction (CSC), a computational method that corrects for the effect of off-targeting on gRNA depletion. We benchmark CSC with data from the Cancer Dependency Map and show that it significantly improves the overall sensitivity and specificity of viability screens while preserving known essentialities, particularly for genes targeted by highly promiscuous gRNAs. We believe this tool will further enable the functional annotation of the genome as it represents a robust alternative to the traditional filtering strategy of discarding unspecific guides from the analysis. CSC is an open-source software that can be seamlessly integrated into current CRISPR analysis pipelines.

## Abstract (web summary)

Off-target effects often confound the interpretation of CRISPR screens. Here, the authors introduce a computational method that corrects for off-targets in gRNA depletion assays, circumventing the need to completely discard unspecific guides.

## Introduction

High-throughput loss-of-function screens can help catalog loci essential to cellular fitness^1–4^ and have been leveraged to systematically identify cancer vulnerabilities that can be exploited therapeutically^1^. The CRISPR-Cas9 genome editing system has become instrumental in these efforts, owing to the ease at which null alleles can be generated in a multiplex manner in both coding and non-coding regions.

Nevertheless, measurements of cellular fitness in CRISPR loss-of-function screens can be confounded by off-target cleavage because gRNAs that lead Cas9 to cleave multiple loci can trigger a DNA-damage response that includes cell cycle arrest^5^. The consequences of off-target cleavage on screen performance have been best characterized for gRNAs targeting amplified genomic regions^6,7^. However, they have also been documented for unspecific gRNAs within published genome-wide libraries both when they have perfect alignment or single mismatches to off-target sites^8^. To minimize the confounding effects of off-targets, unspecific gRNAs are typically identified and discarded during library design. In addition, guides suspected of off-target activity that were unintentionally included in the libraries are further removed from the analysis through the implementation of filters^2,6^. Both these filtering steps are reasonable strategies for screens to protein-coding genes, but they represent major obstacles to the implementation of fitness screens to genomic features that cannot be targeted by specific gRNAs^9^. These include a large fraction of non-coding regulatory elements^9^. Indeed, recent work reported that off-target activity caused the majority of fitness effects in CRISPR essentiality screens to CTCF binding sites^9^, indicating that gRNA specificity is a key confounder in this setting. However, removing unspecific gRNAs at the library design step would render a large fraction of those sites—as well as binding sites for numerous transcription factors—untargetable^9^. Thus, despite the ongoing efforts to comprehensively discover and annotate genomic features^10,11^, a large fraction of them cannot currently be screened for essentiality using conventional CRISPR strategies. This limitation significantly hinders our ability to gain insight into the functional roles of large segments of the genome. It also argues for the need for approaches that can be used as alternatives to filtering unspecific gRNAs, analogous to those used for gRNAs targeting amplified genomic regions^12,13^.

Here, we build upon previous work^9^ by developing a computational method that identifies and corrects for the confounding effect of gRNA off-targeting in high-throughput CRISPR fitness screens. We apply our CRISPR Specificity Correction (CSC) algorithm to genome-wide fitness screens performed by the Cancer Dependency Map initiative^1,2,12^, allowing us to use gold-standard sets of essential and non-essential genes to benchmark our approach^14^. We find that CSC significantly improves screen performance across all cellular lineages, beating gRNA filtering strategies in its ability to discriminate between known essential and non-essential genes. Correction of depletion data with CSC also captured previously missed gene dependencies, even for genes targeted by highly unspecific gRNAs. We believe this tool will further enable the comprehensive functional characterization of coding and non-coding elements in the genome by expanding the set of usable gRNAs in CRISPR libraries. We package CSC as an open-source Python software which we make freely available to the community.

## Results and discussion

**Fig. 1:**
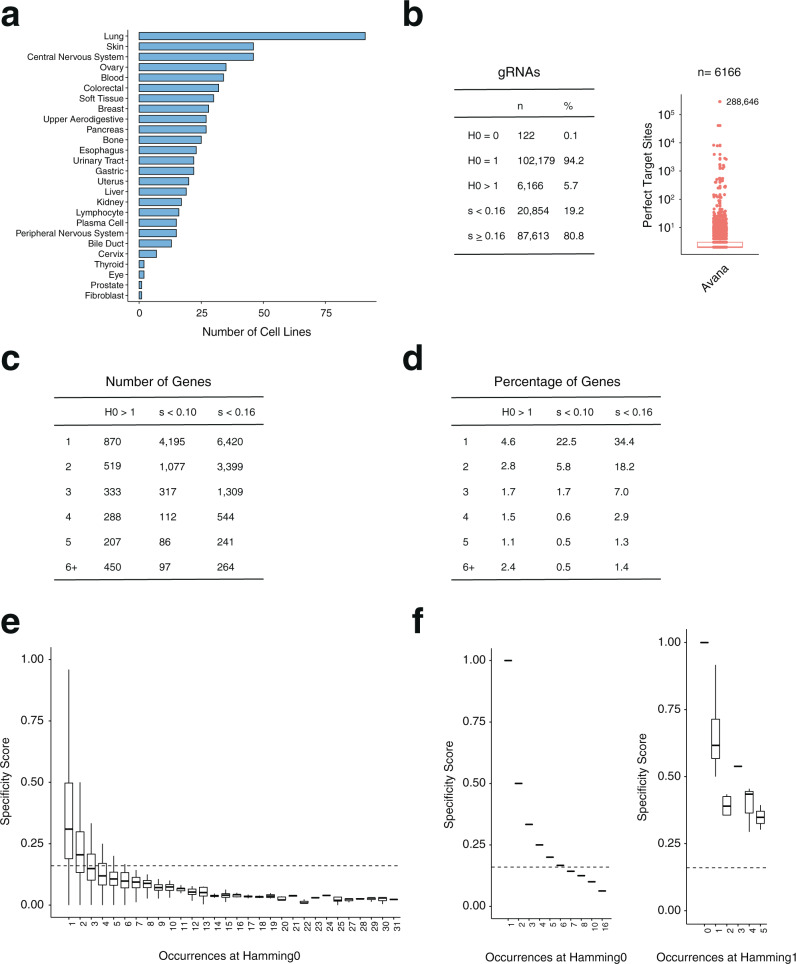
Analysis of the 19Q4 Avana dataset from the DepMap initiative. **a** Number of screened cell lines in each of the 26 lineages represented in the dataset. **b** Left, number and percentage of gRNAs in the Avana library that have 0, 1, or more than 1 perfect targets (H0) in the human genome (hg38 assembly) or that have specificities (*s*) lower or equal/higher than 0.16. Right, number of perfect target sites for gRNAs with H0 > 1. The gRNA in the Avana library with the highest number of perfect targets sites is highlighted. **c**–**d** Summary of the number (**c**) and percentage (**d**) of genes targeted by increasing number of promiscuous gRNAs (1-6+). Promiscuity is defined at three distinct thresholds (H0 > 1, *s* < 0.10, *s* < 0.16). **e** Specificity scores of all gRNAs (*n* = 108,345) with varying numbers of perfect target sites in the genome. *X*-axis is truncated at 31 for simplicity. Dashed line highlights the position of the 0.16 threshold. **f** Specificity scores of gRNAs with varying number of perfect (left; *n* = 2423) or single mismatched (right; *n* = 2374) targets in the genome in the absence of any additional Hamming neighbors. gRNAs with no perfect target in the hg38 genome were excluded from the analysis in **e** and **f**. All boxplots show minimum, maximum, median, first, and third quartiles.

We set out to develop a computational strategy to correct for the effect of off-targeting on gRNA depletion without the need to filter out unspecific gRNAs. To validate our strategy, we focused our analysis on loss-of-function screens from the 19Q4 release of the Project Achilles Avana dataset from the Cancer Dependency Map initiative^2,12^, performed across 26 distinct cellular lineages (Fig. 1a). This dataset represents a useful proof-of-principal scenario as previous work described the presence of unspecific gRNAs in Avana^8,15^ and showed that these unspecific guides confound the analysis of essentiality and contribute to false-positives hits in negative selection screens^8^. As important, the Avana genome-wide library targets gold-standard sets of curated essential and non-essential genes^14^. These gene sets are commonly used to evaluate the performance of CRISPR tools because gRNAs that target them can be considered true-positives and true-negatives in the context of viability screens, since gRNAs that disrupt essential genes are expected to drop out from the population of infected cells over time, while the abundance of gRNAs that disrupt non-essential genes is expected to remain unchanged.

**Fig. 2:**
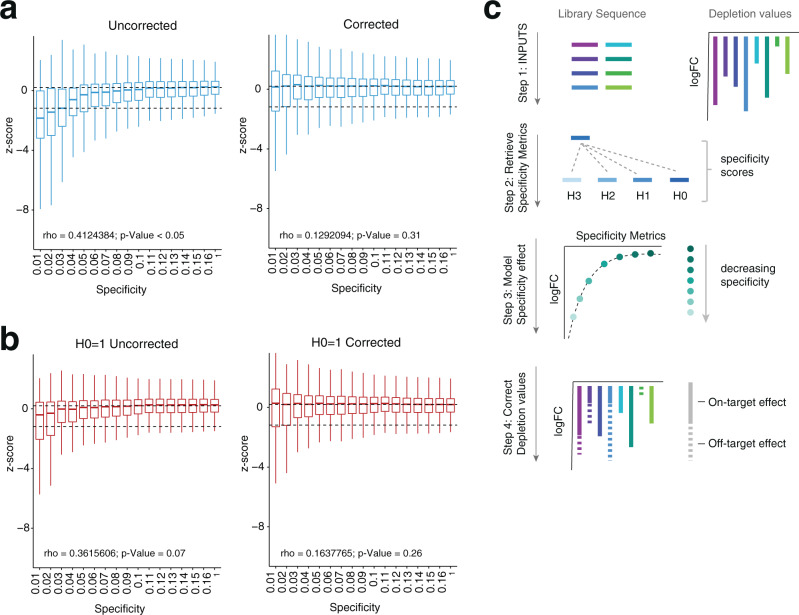
Computational correction of off-target mediated gRNA depletion from CRISPR-Cas9 essentiality screens. **a** Boxplots of *z*-scores for gRNA log2-fold changes for Avana Project Score 19Q4 screens (*n* = 689) across multiple specificity bins before (left) and after (right) correction. Specificity values correspond to highest value of each bin. Dashed lines indicate median depletion of specific gRNAs (GuideScan score = 1) targeting known non-essential (top) or essential (bottom) genes. One-sided Pearson correlation values between specificity and mean depletion of gRNAs in each bin are shown, along with their significance. Boxplots show minimum, maximum, median, first, and third quartiles. **b** As in **a** but plotting only gRNAs that have a single perfect target site in the genome (H0 = 1; H0, hamming distance of 0). One-sided Pearson correlation values between specificity and mean depletion of gRNAs in each bin are shown, along with their significance. Boxplots show minimum, maximum, median, first, and third quartiles. **c** Schematic representation of CSC. Briefly, the correction takes as input the gRNA sequences and corresponding depletion values. For each of the gRNAs, CSC enumerates all off-targets up to Hamming distance of 3 (H3) and computes its specificity score. CSC then uses the off-target information and depletion values to model the effect of off-targeting on gRNA abundance. Finally, it outputs both the corrected depletion values and the specificity metrics for each of the gRNAs. logFC, log fold change.

To systematically evaluate the effect of specificity on gRNA depletion, we enumerated all potential off-targets—between Hamming distances of 0 and 3—for each gRNA in the Avana library using GuideScan^16^. GuideScan is a retrieval-tree-based algorithm that outperforms Bowtie-based tools in the identification of off-target loci^16^, providing an exact and direct enumeration of the potential target space of a gRNA within a user-specified number of mismatches to the guide sequence (Supplementary Note 1 and accompanying Supplementary Table 1, Supplementary Data 1, Supplementary Data 2). Our catalog of potential off-target loci for Avana using this approach surpassed the original off-target estimates reported for this library^15^ as well as the estimates used by Project Achilles in the DepMap data processing pipeline^2^ (Supplementary Data 2, Fig. 1b–d, Supplementary Note 1). To summarize the specificity of each gRNA in this library, we further computed GuideScan’s specificity score. This score aggregates Cutting Frequency Determination values (or CFD, describing the likelihood of an off-target being cut by Cas9 based on the number, position, and identity of mismatches to a 20 nucleotide (nt)-long gRNA)^15^ for all potential target sites enumerated by Guidescan^16^, so that the most specific targeting gRNAs receive a score of 1 and the most unspecific a score of 0 (Fig. 1e, f). In agreement with previous studies^8,9^, gRNAs with low specificities were on average more depleted from the population during viability screens, often beyond the levels observed for gRNAs targeting known essential genes (Fig. 2a, left, Supplementary Fig. 1a–d). This observation held true even for guides that had a single perfect target site in the genome (H0 = 1) but increasing numbers of off-targets with mismatches (Fig. 2b, Supplementary Fig. 1c). Of note, when we looked at gRNAs targeting known non-essential genes^14^—whose representation in the library should remain unchanged over the course of the screen—we found that gRNAs with specificity scores below 0.16 were significantly depleted compared to highly specific guides (specificity score = 1; Kolmogorov–Smirnov test, adjusted for multiple testing). Fold-change distributions of gRNA with a specificity score equal or above 0.16, however, were indistinguishable from those of highly specific guides suggesting that above this threshold the effect of off-target cutting on the guide’s representation in the library is statistically minimal (Supplementary Fig. 1a, Supplemental Note 2).

To determine the extent to which off-target mediated gRNA depletion acted as a confounder in the Achilles dataset, we calculated Bayes Factors (BF) for each gene in individual screens^17^. In this context, BF are an assessment of gene essentiality, with positive values indicating a gene is essential and negative values indicating a gene is non-essential. Gene Set Enrichment Analysis (GSEA) showed that genes targeted by unspecific gRNAs were significantly enriched in high BF values, particularly as the number of unspecific gRNAs per gene increased or as the specificity of the gRNAs that target each gene decreased (see Supplementary Fig. 1e–g for an example cell line). This suggests that, in agreement with previous reports^8,9^, off-targeting may contribute to false-positive dependencies even when multiple independent gRNAs per gene are present in a library. Taken together, these data also validate the Avana dataset from Project Achilles as a suitable model to test our CRISPR Specificity Correction (CSC) algorithm for its ability to correct the confounding effect of off-targeting on gRNA depletion.

CSC takes as inputs the sequence and depletion values of all gRNAs in a screen (Fig. 2c). As a first step, it uses the sequence information to retrieve for each guide the number of potential target sites it has at zero (H0), one (H1), two (H2), or three (H3) Hamming distance to the gRNA sequence as well as their GuideScan specificity score. Although cleavage of sites with a Levenshtein distance of 1 to the gRNA—often referred to as ‘bulged’ sites—has also been reported^18,19^, these types of mismatches rarely preserve Cas9 activity^15^ and therefore were not considered. To retrieve off-target information for each guide in a library, CSC accesses hash tables whose keys contain every Cas9 gRNA that can target the human (hg38) or mouse (mm10) genomes. In the tables, each of these keys is mapped to the gRNA’s specificity metrics which have been previously computed using GuideScan^16^. CSC then uses the five specificity metrics as covariates in a model to assess the contribution of off-target parameters to gRNA depletion via a multivariate adaptive regression spline (EARTH, see Methods). Piecewise linear spline functions, have been previously used to model the effect of genome copy-number alterations on gRNA depletion as they can model nonlinearities and are well suited to deal with the saturating effect of high numbers of cut sites^12^. The EARTH model utilized by CSC has the advantage of automating both the partitioning of the data through its hinge functions as well as the variable selection step, thus providing a flexible approach for off-target correction. In addition, the backward pass improves generalizability of the model and minimizes overfitting of the training data by pruning the least effective terms and selecting the best performing model through generalized cross validation (see Methods for more details on model selection).

Specificity-corrected depletion values for each guide are outputted along with the enumeration of off-target sites and the gRNA’s GuideScan specificity score (Fig. 2c). Importantly, specificity metrics for all inputted gRNA sequences are provided even in the absence of accompanying depletion values. Thus, CSC not only allows users to correct off-target influence in their screening data but also provides direct access to GuideScan’s specificity metrics for any Cas9 gRNA that targets the human or mouse genome. This allows users to determine the potential target space of a gRNA or set of gRNAs even prior to a screen and may further aid CRISPR library design.

**Fig. 3:**
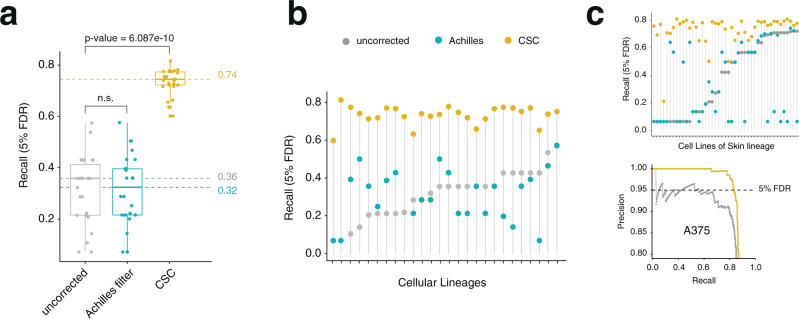
CSC improves the performance of CRISPR-Cas9 essentiality screens. **a** Boxplot showing recall values at 5% FDR for the 19Q4 Project Achilles dataset (*n* = 689) before correction (grey), with the Achilles filter (blue), or with the CSC correction (yellow). Each dot represents the median recall value of a lineage (*n* = 26). Minimum, maximum, median, first, and third quartiles are shown. *p*-values were calculated using a two-sided Wilcoxon test. **b** Median recall values at 5% FDR for each lineage. **c** Top, example plot for a single lineage (skin). Bottom, example precision-recall plot for a single cell line (A375 melanoma cells). Lollipop graphs are plotted by increasing values of the uncorrected pipeline. The maximum recall value at 5% FDR was used for each comparison.

To validate CSC, we applied it to all screens from the DepMap 19Q4 Achilles dataset. As predicted, CSC removed the correlation between gRNA specificity and gRNA depletion (Fig. 2a, b). Additionally, when inferring gene essentiality in each cell line of the Achilles dataset, we found that correction of off-target mediated gRNA depletion by CSC significantly increased both the recall of constitutive essential genes at 5% False Discovery Rate (FDR) of constitutive non-essential genes (Fig. 3) as well as the Area Under the Curve (AUC) of precision-recall curves (Supplementary Fig. 2a, b). Both these metrics evaluate the success of predicting true gene essentialities. This trend remained true when looking at aggregate data for the entire Achilles data set (Fig. 3a), as well as at the level of individual lineages and cell lines (Fig. 3b, c, Supplementary Fig. 3). Of note, CSC markedly outperformed the filtering strategy implemented by Project Achilles to deal with unspecific gRNAs^2^ (Fig. 3, Supplementary Fig. 3b).

**Fig. 4:**
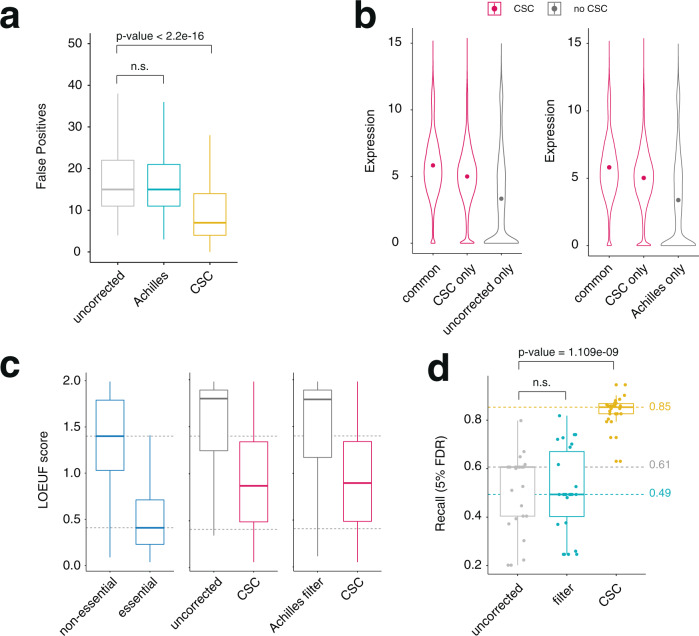
CSC improves the identification of true gene dependencies. **a** Boxplot showing number of false positive hits for the 19Q4 Project Achilles dataset (*n* = 689) before correction (grey), with the Achilles filter (blue), or with the CSC correction (yellow). For comparison purposes, Bayes Factor thresholds were varied to return the same number of hits across all three pipelines. *p*-values were calculated using a two-sided Wilcoxon test. **b** Violin plots showing the expression levels (log2(TPM+1)) of genes in the cell lines in which they were identified as dependencies. Left graph shows genes identified both before and after correction (common), only after correction (CSC only) or only before correction (uncorrected only). Right graph shows genes identified as dependencies both in pipelines that implement CSC and Achilles (common), or only in one of the pipelines (CSC only, Achilles only). Dot represents the mean value. **c** Boxplots showing the LOEUF scores for genes identified as dependencies by only one of the pipelines. Exclusive dependencies are defined as genes that score in more than 15 cell lines in one pipeline and none in the other (uncorrected versus CSC: *n* = 832; Achilles versus CSC plot *n* = 835). LOEUF scores for constitutive essential and non-essential genes are shown on the left as a reference (*n* = 1169), and their median values are highlighted across all plots with a dashed line. **d** Recall values as in Fig. 2a but calculated using known constitutive essential and non-essential genes targeted by at least one unspecific gRNA (H0 > 1). Filter corresponds to a pipeline where gRNAs with more than one perfect target site are removed before the analysis. Boxplots show minimum, maximum, median, first, and third quartiles.

With the increased recall at 5% FDR, we observed a concomitant increase in the number of genes identified as essential. In total, 12,444 genes scored as a dependency in at least one screen when CSC was implemented, compared with 5831 and 6018 genes for data not corrected for off-targeting or when unspecific gRNAs were removed with the Achilles filter, respectively. To determine if these results constituted true gene essentialities, we first looked at the occurrence of known false positives. Because the number of false positives is influenced by the total number of genes identified as hits, we varied Bayes Factor thresholds for each screen to yield the same number of hits across each pipeline as previously described^20^. Then, for each screen we counted the number of gold-standard non-essential genes^14^ that had scored as hits. We found that CSC led to a significant reduction in the number false positives compared to uncorrected data, again outperforming the filtering strategy implemented by project Achilles (Fig. 4a). At this fixed number of positive hits, CSC also led to a significant increase in both precision and recall (Supplementary Fig. 2c). We then examined the expression levels of genes inferred as essential by each of the three analysis pipelines. We found that those genes identified as essential after off-target correction by CSC tended to be well expressed in the cell line in which they scored as hits (Fig. 4b, Supplementary Fig. 4a). By contrast, for each screen, genes scoring as essential in uncorrected data but not in data corrected with CSC tended to have significantly lower expression levels in the respective cell lines. In fact, a substantial subset of these genes was below bulk RNA-seq detection threshold, suggesting that they may represent false-positive hits. Similarly, genes identified as essential when data was corrected with the Achilles filter but not when data was corrected with CSC tended to be lowly expressed in the cell lines they scored as hits or below detection threshold, suggesting these may also represent false positives (Fig. 4b, Supplementary Fig. 4a).

Next, we looked for evidence of functional essentiality for genes identified as putative dependencies. Essential genes are under evolutionary pressure to maintain sequence integrity, and mutations that inactivate their functions are expected to be depleted from natural human populations. In contrast, non-essential genes whose disruption does not affect organism health or function, are expected to tolerate the accumulation of inactivating mutations. As such, we took advantage of the Genome Aggregation Database (gnomAD)^21^, which catalogs high-confidence predicted loss-of-function variants and uses these to classify human genes according to the mutational constraint they are under. Specifically, the LOEUF score places genes along a spectrum of tolerance to inactivating mutations, where genes that play essential cellular roles, and therefore are under high mutational constraint, receive low LOEUF scores, while genes whose disruption has no impact on cell viability or organismal health and are therefore under low mutational constraint in the human population receive high scores^21^. We confirmed that these scores can reflect gene essentiality by applying them to the curated sets of essential and non-essential genes^14^ (Fig. 4c, left). We then retrieved LOEUF scores for genes consistently identified as essential by one analysis pipeline (defined as genes that score as hits in more than 15 distinct cell lines) but not the other. We found that genes consistently identified as essential in data corrected using CSC but that did not score as essential in any screen when using uncorrected data or data corrected using the Achilles filter tended to have low LOEUF values. On average these scores were well below those attributed to constitutive non-essential genes (Fig. 4c). This suggests that genes exclusively identified as dependencies after correcting off-targeting with CSC are under mutational constraint in the human population and therefore presumably play essential roles in human cells. In contrast, genes that scored in more than 15 distinct screens only before data correction or only after removing promiscuous gRNAs through the Achilles filter—but that did not score as hits after data was corrected for off-targeting using CSC—tended to have higher LOEUF values, often above those of constitutive non-essentials. These results suggest that their inactivation may be well tolerated in humans. Taken together, these data are consistent with the notion that genes identified after computational correction by CSC reflect true essentialities, and that CSC implementation minimizes the occurrence of false-positive hits. It also suggests that CSC outperforms the current filtering approach implemented by Project Achilles to deal with unspecific gRNAs (Fig. 4a–c).

To test CSC’s performance in the context of highly unspecific libraries, we selected only genes that are targeted by at least one gRNA containing multiple perfect target sites in the human genome (H0 > 1) with the Avana library. The resulting subset of gRNAs approximates better the low specificity of libraries designed to target non-coding regulatory elements in the genome^9^ (Supplementary Fig. 4b, c), while still targeting true-positive and true-negative genes that can be used to benchmark CSC. The low specificity of gRNA libraries cutting within non-coding motifs stems from the fact that predicted *cis*-regulatory elements are typically small, with lengths several fold below those of average exons. As a result, the traditional approach used for screens of protein-coding genes, where known unspecific gRNAs are excluded at the step of library design, is not viable for a large fraction of regulatory sequences. Indeed, filtering out gRNAs suspected of off-target activity would render many of these regulatory sequences untargetable^9^ (Supplementary Fig. 4d).

We used this pool of unspecific Avana gRNAs to reanalyze all screens from the DepMap 19Q4 dataset and re-calculate precision and recall curves. As expected, in the context of this highly unspecific set of gRNAs, the simple filtering of known promiscuous guides was an ineffective strategy to correct off-targeting (Fig. 4d). By contrast, we found that correction of off-target mediated gRNA depletion by CSC substantially increased recall (5% FDR) across all lineages of the dataset (Fig. 4d). This suggests CSC can help retrieve known gene dependencies even from highly promiscuous gRNA pools.

**Fig. 5:**
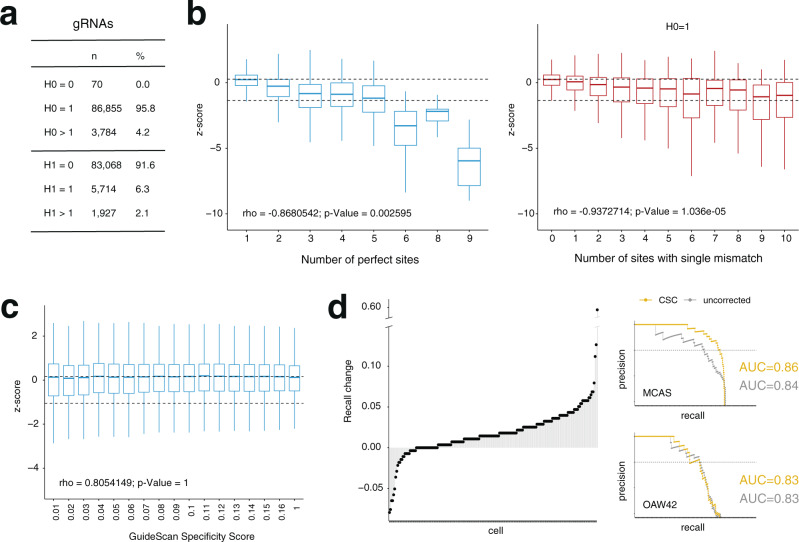
Analysis of the Sanger dataset from Project Score. **a** Number and percentage of gRNAs in the Sanger library that have 0, 1, or more than 1 perfect targets (H0) in the human genome (hg38 assembly) or that have 0, 1, or more than 1 targets with a single hamming mismatch (H1). **b** Left, boxplots of *z*-scores of gRNA log_2_FC for all Project Score screens across multiple H0 bins (i.e., increasing numbers of perfect targets). Right, boxplots of *z*-scores for gRNA log_2_FC for all Project Score screens across multiple H1 bins (i.e., increasing numbers of targets with a single hamming mismatch) for gRNAs that have a single perfect targeting the genome (H0 = 1). Dashed lines indicate median depletion of specific gRNAs (H0 = 1, H1 = 0) targeting known non-essential (top) or essential (bottom) genes. One-sided Pearson correlation values between mean depletion and number of off-targets, as well as the significance of the correlation are shown below. *n* = 324. **c** As in **b** but plotting *z*-scores of gRNA log_2_FC against GuideScan’s specificity bins. Specificity values correspond to highest value of each bin. Note how for 19-mer gRNAs there is no correlation between GuideScan’s score and depletion. One-sided Pearson correlation values between mean depletion and number of off-targets, as well as the significance of the correlation are shown below. *n* = 324. **d** Left, change in recall (5% FDR) for each Project Score screen after being corrected with CSC. On the right we show examples of precision-recall curves for screens showing increased recall (MCAS, second most improved) or decreased recall (OAW42, second most decreased) after correction. Dashed line highlights 0.95 precision value (which corresponds to a false discovery rate of 5%). Note how the improved recall in the MCAS screen is accompanied by an increase in the area under the curve (AUC). In contrast, the decreased recall in the OAW42 screen is accompanied by no change in the AUC. All boxplots show minimum, maximum, median, first, and third quartiles.

To test the generalizability of CSC, we next applied it to the genome-wide screens released by Project Score^1^ (Fig. 5). This dataset was generated using the Sanger genome-wide library^22^, which was designed to contain gRNAs with 19 nt-long complementarity to the genome and therefore follows distinct off-target rules than those established for the more common 20-nt design. Like Avana, the Sanger library contains a non-negligible fraction of gRNAs with perfect (H0 > 1) or near perfect (H1 > 0) off-targets (Fig. 5a, Supplementary Data 3). In addition, as described for Project Achilles screens, gRNAs with increasing numbers of perfect target sites were increasingly depleted from essentiality screens released by Project Score (Fig. 5b, left). This was also true for gRNAs with only one perfect target site in the genome (H0 = 1) but increasing numbers of off-targets with single-mismatches (Fig. 5b, right). This suggests that even in the context of a 19-nt gRNA design, cleavage of mismatched off-targets can cause measurable toxicity to the cells. In contrast to the Achilles dataset, we found no correlation between GuideScan’s Specificity score and gRNA depletion in Project Score screens (Fig. 5c). This is expected as this score is computed using the CFD metric which was empirically developed using 20 nt-long gRNAs. Thus, the Project Score dataset provides a unique challenge for CSC and an opportunity to test its flexibility in correcting off-target mediated gRNA depletion in CRISPR viability screens based on distinct gRNA designs. Applying CSC to Project Score screens resulted in correction models that included both H0 (number of Hamming 0 targets) and H1 (number of Hamming 1 targets) as the main covariates, in stark contrast to models generated on Avana data where GuideScan’s specificity score was consistently the most important covariate (compare example metric output files for each dataset, provided as Supplementary Data 4 and Supplementary Data 5 for an Avana and Sanger screen, respectively). This reflects the weakness of GuideScan’s specificity score in summarizing off-target cleavage by 19-nt gRNAs (Fig. 5c) and highlights the ability of CSC to discriminate the most informative covariates for its correction models. As before, we evaluated the performance of CSC in the Project Score dataset by calculating the recall of known essential genes at 5% FDR (Fig. 5d). We also calculated the AUC for precision-recall curves before and after correction. We found that CSC led to a significant increase in both metrics (recall *p*-value <2.2e-16, AUC *p*-value = 5.58e-8; two-sided, Wilcoxon test). Together, these data demonstrate the generalizability of CSC and its ability to increase the sensitivity of essentiality screens performed with distinct gRNA library designs.

We note that for a small number of Avana and Sanger screens (38 and 26 screens, respectively) correction of off-targeting by CSC led to an apparent drop in recall at 5% FDR (Supplementary Fig. 3 and Fig. 5d). This effect does not appear to be caused by distortions introduced by CSC, as the mean distance between the fold-changes of known essential and non-essential genes is minimally impacted by the correction and is statistically identical between screens where CSC increases or decreases recall (Supplementary Fig. 2d; two-sided Kolmogorov–Smirnov test). In addition, while increased recall is accompanied by a significant increase in the AUC of the corresponding curve (Project Achilles *p*-value <2.2e-16, Project Score *p*-value = 9.404e-09; two-sided, Wilcoxon test), lower recall is not accompanied by statistically lower AUC values (Project Achilles *p*-value = 0.1159, Project Score *p*-value = 0.4186; two-sided, Wilcoxon test). This suggests that the drop in recall at 5% FDR we have documented in a minor fraction of screens represents only a local feature of the curve and not a general trend across all decision thresholds (See Supplementary Fig. 2b and Fig. 5d for representative curves). To further ensure the robustness of the corrections and provide users with full information of how they were generated, CSC outputs for each screen a file describing the model used along with its performance metrics. These include the RMSE of the model (i.e., the square root of the variance of the residuals), a measure of how accurately it predicts the impact of off-targets on gRNA depletion (see Supplementary Data 4 and Supplementary Data 5 for example files). Additionally, CSC allows users to define RMSE thresholds above which no corrections are performed and only off-target descriptions for each gRNA in the library are outputted.

Finally, BAGEL2 is a recently published hit-calling algorithm that includes some degree of multi-targeting correction^20^. BAGEL2 is efficient at gene essentiality calling but it does not segregate the multi-targeting correction and hit-calling functions preventing us from directly comparing it to CSC. However, BAGEL2 uses Bowtie to generate its off-target predictions using settings that can miss perfect and near perfect target sites (see Supplementary Note 1, Supplementary Data 1, and Methods). Additionally, it discards from its analysis gRNAs for which it identifies more than 10 perfect sites or more than 10 sites with a single mismatch, and therefore will have difficulty in handling libraries with particularly unspecific gRNAs, as those that may be used for the screening of non-coding regulatory elements. Additional advantages of CSC that may be particularly useful to users include the full description of the off-target information of every gRNA submitted as an input, as well as information of how off-target metrics were incorporated in the correction model, how well the model performed, and how the correction modified original depletion values. Finally, CSC provides users with means to correct for the confounding effect of off-targets as part of their standard hit-calling pipelines, therefore providing increased flexibility in its employment.

In summary, we present here a flexible computational correction that minimizes the confounding effect of unspecific gRNAs in CRISPR-Cas9 essentiality screens leading to improved sensitivity and reduced false-positive hits in genome-wide screens. We believe CSC will be a powerful aid to ongoing efforts to catalog genomic loci required for cellular fitness, particularly in the context of screens targeting highly repetitive genomic regions—such as non-coding regulatory elements—where the design of specific libraries and gRNA filtering approaches are not feasible^9^. To facilitate its incorporation into existing CRISPR analysis pipelines including those that correct for genomic amplifications (Supplementary Fig. 2e, see Methods), we make the software freely available as a Python package at https://bitbucket.org/arp2012/csc_public/src/master/.

## Methods

### Screening data

Raw read counts of CRISPR viability screens (Broad DepMap project 19Q4) performed with the Avana library were downloaded from the DepMap project data repository (https://figshare.com/articles/DepMap_19Q4_Public/11384241/2). Raw read counts of CRISPR essentiality screens (Sanger, release 1, 5^th^ April 2019) performed with the Sanger library were downloaded from the Project Score page (https://score.depmap.sanger.ac.uk/downloads).

### Guide RNA preprocessing

Guide RNAs with less than 30 reads in the initial plasmid counts were removed. Counts for all screen replicates and corresponding plasmid library were adjusted by median-ratio normalization to account for the effect of library sizes and read count distributions. Finally, for each screen, log_2_-fold changes for individual gRNAs were calculated between the initial plasmid library counts and the post-screen counts for each replicate experiment. The mean log_2_-fold changes between replicates was used as the final log_2_-fold changes value for each gRNA.

### Off-target data

We downloaded the sequence and annotation data for the hg38 assembly of the human genome from the UCSC database^23^ and used it to construct a retrieval tree (trie) consisting of all possible 20mer Cas9 gRNA target sites in the human genome with the GuideScan software^16^. In contrast to the original trie^16^, this retrieval tree was constructed without inclusion of alternative chromosome data, so that they did not artificially inflate the enumeration of off-targets. To determine the mismatch neighborhood for each gRNA in the Avana library, we traversed their sequences through the trie to exhaustively determine all neighbors up to and including Hamming distances of 3. Specificity scores for each gRNA were computed using Hamming distance neighbors using our previously described strategy^16^, which incorporates Guidescan’s ability to faithfully enumerate all potential target sites up to a specified number of mismatches, with CFD score’s prediction of how likely each of those sites is to be cut^15^. All these metrics are used as covariates (*x*) in the CSC model (see below).

### Gene set enrichment analysis

GSEA was performed using the FGSEA R package, Release 3.13. and available at: http://bioconductor.org/packages/release/bioc/html/fgsea.html.

Enrichment and *p*-values were calculated against 100,000 random gene sets.

### Model comparison and selection

We tested various machine learning models including a linear model lasso with least angle regression, random forest regression, and multivariate adaptive regression splines (EARTH) by regressing the mean depletion values of each gRNA against its GuideScan’s specificity score and the number of mismatch neighbors at Hamming distances 0, 1, 2, and 3. We modeled the impact of specificity on gRNA depletion on all screens from the 19Q4 Project Achilles dataset. For each screen we used 90% of the data for training with 10% being held out for testing. The mean squared error of these models was significantly lower with the multivariate adaptive regression splines (Supplementary Fig. 5). Because EARTH performed best out of the ensemble of regressors tested and automatically feature engineers non-linearities and interaction terms using the input covariates it was selected as the base model for CSC.

### Multivariate adaptive regression splines

We developed a model that assumes that the measured depletion value (*D*) of a gRNA (*i*) in any individual screen is the sum of gene-knockout effects (*G*_*i*_) and off-target effects (*O*_*i*_).

1 $${D}_{i}={G}_{i}+{O}_{i}$$

To estimate *O*_*i*_, we use Multivariate Adaptive Regression Splines^24^ (EARTH) which can model non-linearities in the data as well as interactions between variables. The model takes the form of the following equation

2 $${\hat{O}}_{i}(x)=\mathop{\sum }\limits_{j\,=\,1}^{J}{C}_{j}{B}_{j}(x)$$

where the estimated contribution of off-targeting to gRNA depletion (${\hat{O}}_{i}$) can be approximated by the weighted sum of *J* basis functions *B*_*j*_ derived from the model predictor variables (*x*). *C*_*j*_ are coefficients of expansion whose values are jointly adjusted to give the best fit to the data. The basis functions *B*_*j*_can take the form of (i) a constant 1, which represents the intercept of the model; (ii) a hinge function derived from a predictor variable, or (iii) a product of two or more hinge functions each derived from different predictors to capture their interaction.

### Model training and pruning

The model starts with the intercept term (*B*_0_(*x*) = 1, with intercept at *C*_0_). It then iteratively adds new basis functions in the form of hinge functions or products of hinge functions. At each step, the new terms are selected and added into the model as to minimize the sum of squared error using ordinary least squares method. This forward pass proceeds until the residual error consistently falls below the stopping threshold (minimal change in mean squared error (MSE) with additional terms). To prevent over-fitting and improve generalization, the forward pass model undergoes a backwards pass, where model terms are removed in a stepwise manner with subsequent reassessment for increases in the sum of squared error obtained in this sub-model. Selection for the optimal sub-model is done using generalized cross-validation (GCV) which optimizes tradeoff between bias and variance. The model with the lowest GCV is selected as the optimal model. An example file detailing model metrics for an Avana and a Sanger screen are provided as Supplementary Data 4 and Supplementary Data 5.

In the development of this model training data consists of 90% of all input data; test data consists of the remaining 10%. Test error was assessed as root mean squared error between the predicted and actual values of test data.

### CSC software and implementation

The CSC was packaged in Python (version 3.8.8) with Avana, Brunello, GeckoV1, GeckoV2, and Sanger libraries as package data. Pickle files for hg38 and mm10 genomes are also provided in a repository, to allow CSC to be implemented for any custom human or mouse libraries based on a 20-mer gRNA design. We also provide a Docker image. The software is also available via PyPi. All these files are freely available to download from our bitbucket repository (see Code Availability).

### Alternative approaches for gRNA off-targeting correction

To benchmark CSC, we compared its performance against the current approach of filtering out gRNAs suspected of off-target activity as implemented by Project Achilles. Information about this filter (which can be downloaded from the DepMap data repository as “Achilles_dropped_guides.csv”) is provided here as part of Supplementary Data 2 (columns 8 and 9). The Achilles filter list was generated from runs of CERES, and includes guides flagged for potential off-target activity by CERES based on being the sole efficacious gRNA for a gene receiving a label of “guide_dropped_by_ceres”. In addition, this file enumerates the estimated number of perfect matches for each guide in the column “Achilles n_alignments”. These alignments are performed with Bowtie against the 20-nucleotide long sequence of each gRNA and subsequently filtered for the presence of PAM sequence motif in the form of NGG^2^. gRNAs that have no perfect alignment to hg38 or that are found to have more than one perfect target site through this method are dropped from the analysis and flagged as “not_aligned” or “in_dropped_guides”, respectively.

As discussed in Supplementary Note 1, and shown Supplementary Data 2, the filtering list that is generated through the method described above significantly underestimates the number of promiscuous gRNAs in the Avana library. In fact, estimation of perfect target sites by this approach only surpasses that of the GuideScan retrieval trie algorithm in 8 cases (Supplementary Table 2).

Manual curation of each of these shows that the additional sites identified by Bowtie but not GuideScan were not adjacent to PAM motifs, and therefore do not represent potential target sites for the guide RNA in question.

In our final analysis to test CSC’s performance in highly promiscuous gRNA pools (Fig. 4d), we compare it with a filter that accurately removes gRNAs with more than a single perfect target site, as identified by GuideScan. The identity of these guides, containing only 1 target site with Hamming distance of 0 (H0) compared to the gRNA, can be found in Supplementary Data 2. Each of these three approaches (CSC, Achilles filter, filter H0 = 1) was integrated within otherwise identical analysis pipelines and compared against a pipeline in which no off-target management was performed.

BAGEL2 is a recently published essentiality classifier that includes off-target correction as part of its algorithm^20^. We were unable to segregate the off-target correction component of this tool from its hit-calling component and therefore could not directly compare it to CSC. However, like the Achilles filter, the off-target enumerations generated and used by BAGEL2 for its corrections are done using Bowtie. As discussed in Supplementary Note 1, this can result in an underestimation of off-targets. We have attempted to compare the off-target description generated by BAGEL2 for the Avana library with that of CSC. However, the BAGEL2 publication does not provide a list of the off-target enumerations for this library used for the multi-targeting correction. Nevertheless, the ‘precalc_library_alignment_info.py’ script deposited on the BAGEL2 GitHub page (https://github.com/hart-lab/bagel) suggests that alignments are performed with Bowtie, using the following parameters:

$$bowtie-v\,3-l\,5-a-x\,BowtieIndex-r\,query.txt-S\,output.txt$$

As we show in Supplementary Data 1, these parameters can miss off-targets at edit distances of 0,1, and 2. In addition, these parameters are unable to retrieve off-targets at edit distances of 3, as N is considered a mismatch by Bowtie, and -v is an integer between 0 and 3. Therefore, these settings do not guarantee exhaustive off-target search within edit distances of 2 and cannot identify off-targets at edit distances of 3, which in our model can still contribute to improper gRNA depletion.

### Evaluation of alignment methods for gRNA off-target search

To compare different alignment methods in their ability to identify gRNA off-targets, we took the sequence of the most unspecific gRNA in the Avana library (TGTAATCCCAGCACTTTGGG) and appended ‘NGG’ at its 3′ end. The resulting 23-nucleotide long sequence represents a potential perfect target sequence for this gRNA and was used as the query sequence for all alignment tools, using the UCSC hg38 genome assembly. These tools include BLAT^25^, Bowtie^26^, Bowtie2^27^, STAR^28^, and BWA^29^. In Supplementary Data 1, we show the total number of alignments obtained with each of these tools and the parameters used in the corresponding run. To identify which of these alignments corresponded to possible target sites for this gRNA, we extracted the sequences corresponding to each alignment coordinate and filtered them based on the presence of an intact NGG PAM, to remove alignments in which mismatches disrupted this motif. We classified alignments with an intact PAM based on their edit distance to the gRNA sequence, using the NM:i:* tag and taking into consideration whether the alignment tool considered ambiguous characters mismatches.

### Precision-recall analysis

To benchmark the performance of CSC, we generated precision-recall curves for all screens after being processed through each analysis pipeline, using the set of constitutive essential and non-essential genes defined in Hart et al.^14^, as references. Precision and Recall were calculated as:

3 $$Precision=TP/(TP+FP)$$

4 $$Recall=TP/(TP+FN)$$

Where: *TP* (True Positives) is the number of positive hits from the gold-standard essential gene set, *FP* (False Positives) is the number of positive hits from the gold-standard non-essential gene set, and *FN* (False Negatives) is the number of gold-standard non-essential genes that did not score as hit. Briefly, these gold-standard sets of genes were defined in ref. ^14^ by selecting genes that emerged as essential in shRNA screens in 72 different cell lines, and then filtering for genes that are constitutively and invariantly expressed across both the ENCODE and the Illumina BodyMap RNA-seq datasets. Reference non-essential genes were selected as those probed in the shRNA screens with no evidence for impact on cell growth and that in addition were not expressed (< 0.1 FPKM) in the majority of tissues and cell lines tested (15/16 BodyMap tissues; 16/17 ENCODE cell lines). Since they were first defined, these lists of ‘constitutive essential and non-essential genes’ have been further validated by multiple independent approaches.

Precision-Recall Curves were generated based on ordered BF values (see below). The BF value corresponding to 95% of precision (meaning the value for which at least 95% of genes are known essentials) was taken as the 5% False Discovery Rate threshold (FDR = 1 - precision). The percentage of reference essential genes identified as essential at that BF threshold was taken as the recall value at 5% FDR. In cases where multiple threshold values had a precision of 95%, that corresponding to the highest recall value was used. Area Under the Curve (AUC) was calculated using the ‘PRROC’ R Package (version 1.3.1)^30^.

### Inference of gene essentiality

The Bagel software (version 0.91)^17^ was used to infer gene essentiality based on log_2_-fold changes of gRNAs for each gene. This software uses a supervised learning method which implements Bayesian statistics and outputs for each gene a Bayes Factor (BF) value based on the likelihood that the observed fold-changes of the gRNAs that target it were drawn from reference essential or non-essential distributions^17^. For each screen, essential genes at 5% FDR we identified by selecting those with BF values above the threshold identified in the precision-recall analysis.

### Distortion metric

We quantified the distortion introduced by CSC for each screen by calculating the Mean and Median Distortion^31^.

### Gene expression data

RNA-seq TPM gene expression data (log_2_-transformed using a pseudo-count of 1) for protein coding genes was downloaded from the DepMap project data repository (https://figshare.com/articles/DepMap_19Q4_Public/11384241/2).

### Genome annotations

Binding site predictions for miRNAs expressed by the *miR-17~92* cluster^32^ were retrieved from TargetScan^33^. Bed files for transcription factor motif archetypes overlapping consensus DNaseI footprints^34^ were downloaded from https://www.vierstra.org/resources/dgf.

### Combined correction of off-targets and copy-number effects

To simultaneously correct for copy number and off-targets effects we ran CRISPRcleanR^13^ on median-ratio normalized reads using the pre-computed files provided for the Avana library. CRISPRcleanR corrected fold-changes were then used to correct for off-target effects using CSC.

### Reporting summary

Further information on research design is available in the Nature Research Reporting Summary linked to this article.

## Supplementary information

Supplementary Information

Description of Additional Supplementary Files

Supplementary Data 1

Supplementary Data 2

Supplementary Data 3

Supplementary Data 4

Supplementary Data 5

Reporting Summary

## Data Availability

The DepMap data used in this study is available at the DepMap project data repository (https://figshare.com/articles/DepMap_19Q4_Public/11384241/2) for screens performed with the Avana library, and at the Project Score page (https://score.depmap.sanger.ac.uk/downloads) for screens performed with the Sanger library. The human genome sequence used to enumerate potential off-target sites was downloaded from the UCSC genome database (https://genome.ucsc.edu/). RNA-seq TPM gene expression data (log2-transformed using a pseudo-count of 1) for protein coding genes can be downloaded from the DepMap project data repository (https://figshare.com/articles/DepMap_19Q4_Public/11384241/2). Binding site predictions for miRNAs expressed by the *miR-17~92* cluster can retrieved from TargetScan. Bed files for transcription factor motif archetypes overlapping consensus DNaseI footprints can downloaded from https://www.vierstra.org/resources/dgf.

## Code availability

Scripts for off-target enumeration and CSC implementation are freely available at our Bitbucket repository (https://bitbucket.org/arp2012/csc_public/src/master/). A PyPi package of the software can be found at pip3 install crispr-CSC.
