# Author Correction: CSC software corrects off-target mediated gRNA depletion in CRISPR-Cas9 essentiality screens

**DOI:** 10.1038/s41467-022-29598-6

**Published:** 2022-04-01

**Authors:** Alexendar R. Perez, Laura Sala, Richard K. Perez, Joana A. Vidigal

**Affiliations:** 1 Laboratory of Biochemistry and Molecular Biology, National Cancer Institute, Bethesda, MD USA; 2 Department of Anesthesia and Perioperative Care, University of California, San Francisco, San Francisco, CA USA; 3 School of Medicine, University of California, San Francisco, San Francisco, CA USA; 4 Department of Anesthesiology, Perioperative and Pain Medicine, Stanford University, Palo Alto, CA USA

**Keywords:** Functional genomics, CRISPR-Cas systems

Correction to: *Nature Communications* 10.1038/s41467-021-26722-w, published online 09 November 2021.

The original version of the Supplementary Information associated with this Article did not include Supplementary Figs. 1–5. The HTML has been updated to include a corrected version of the Supplementary Information.

## Supplementary information

Update Supplementary Information

