# Supplementary material for: CSC software corrects off-target mediated gRNA depletion in CRISPR-Cas9 essentiality screens
Source: Nat Commun. 2021 Nov 9;12:6461. doi: 10.1038/s41467-021-26722-w (PMC8578331; doi:10.1038/s41467-021-26722-w)
Supplement: Supplementary file 1 — Supplementary Information [file 41467_2021_26722_MOESM1_ESM.pdf]

Perez Sup. Fig.1

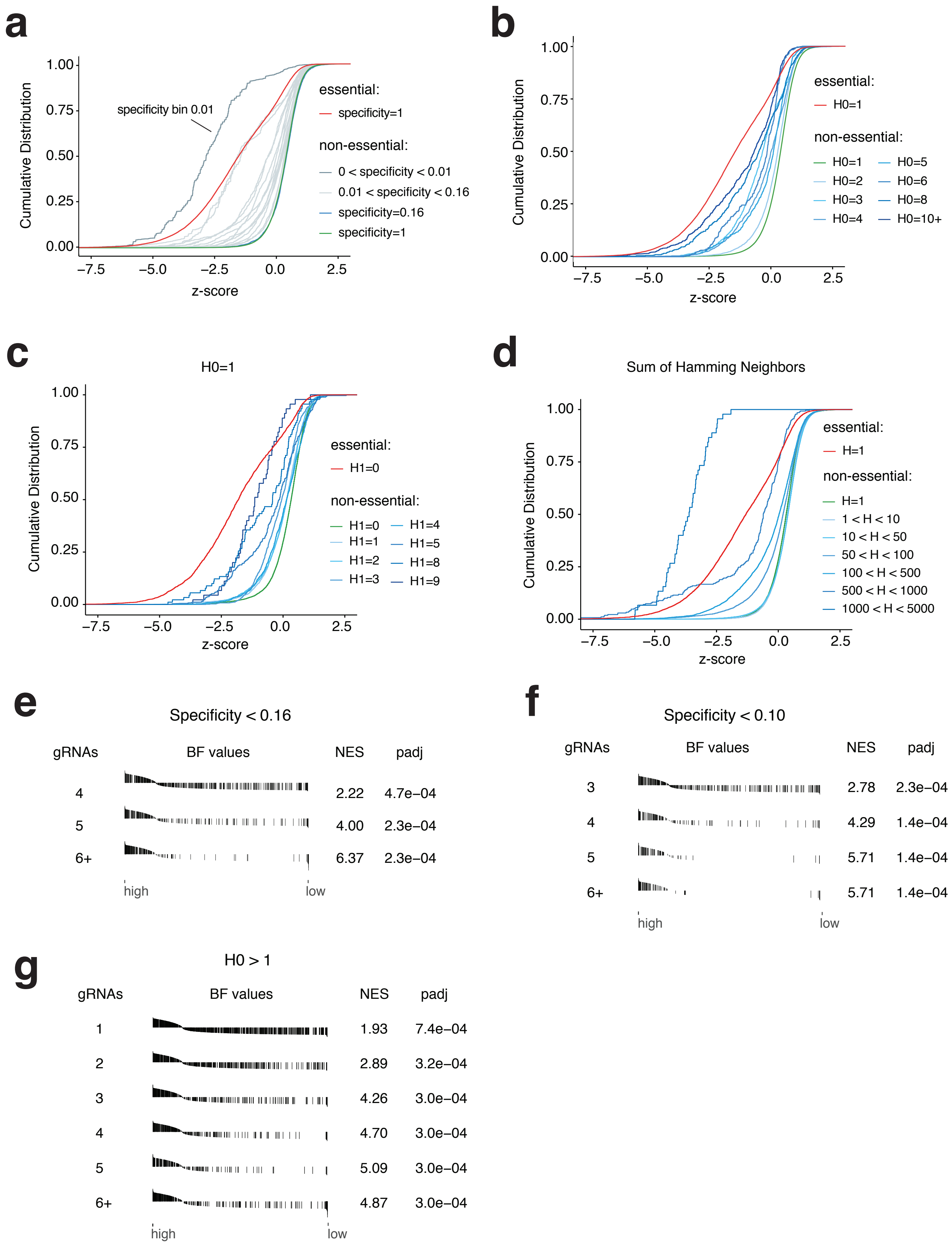

Perez Sup. Fig.2

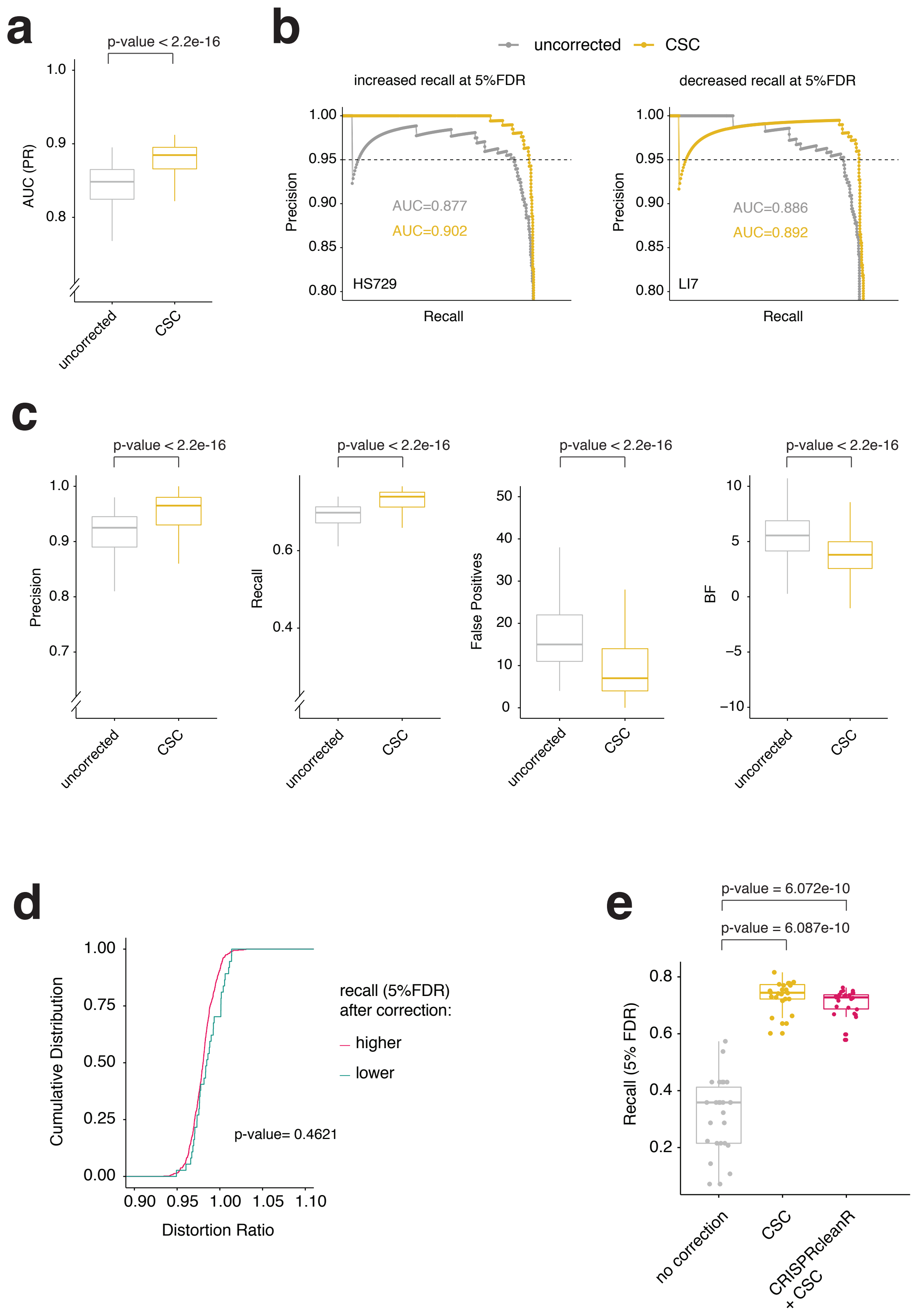

Perez Sup. Fig.3

a

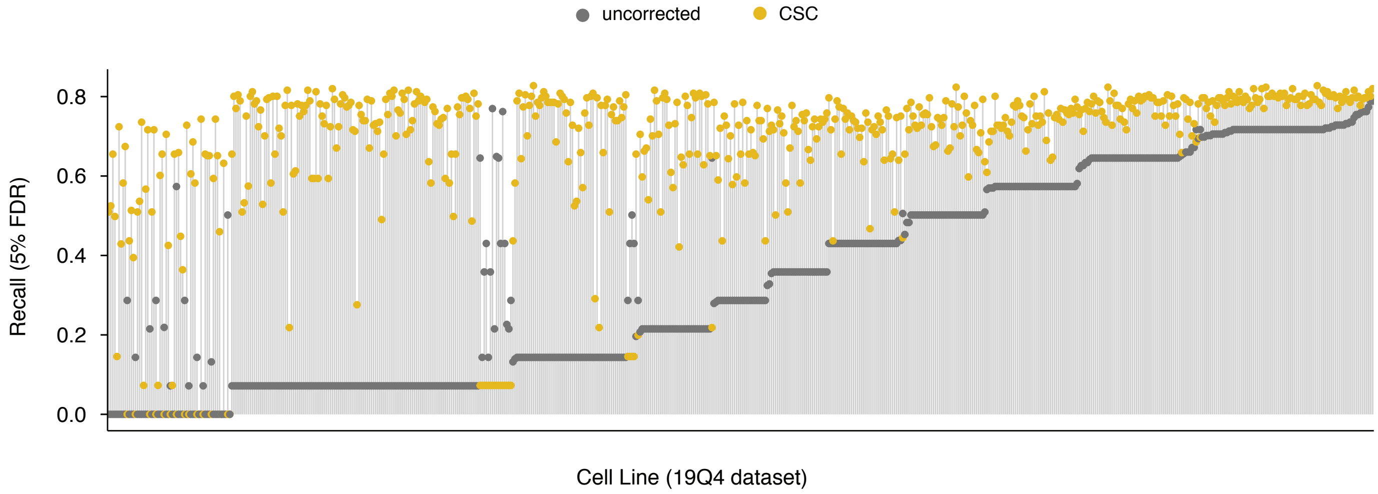

b

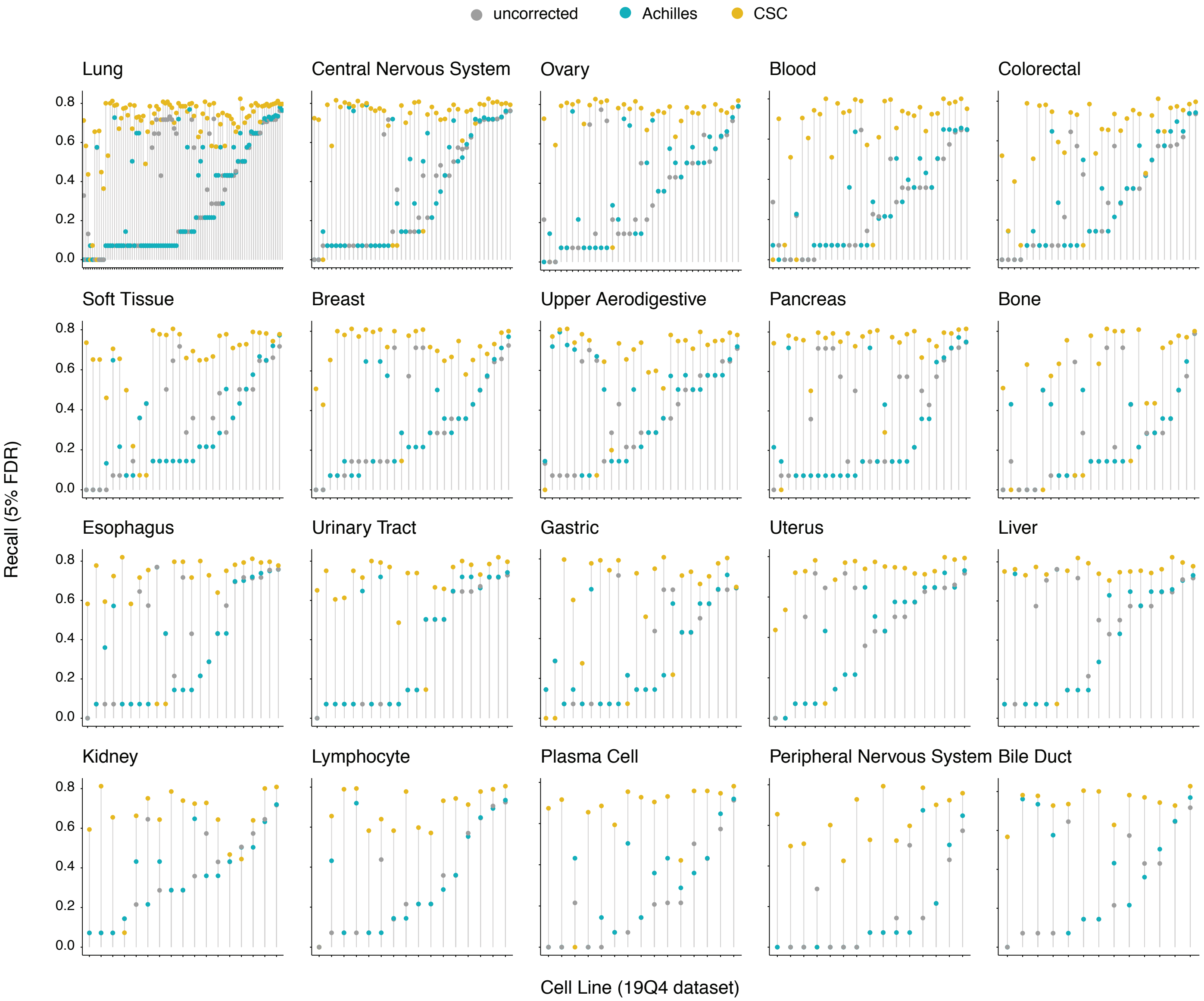

Perez Sup. Fig.4

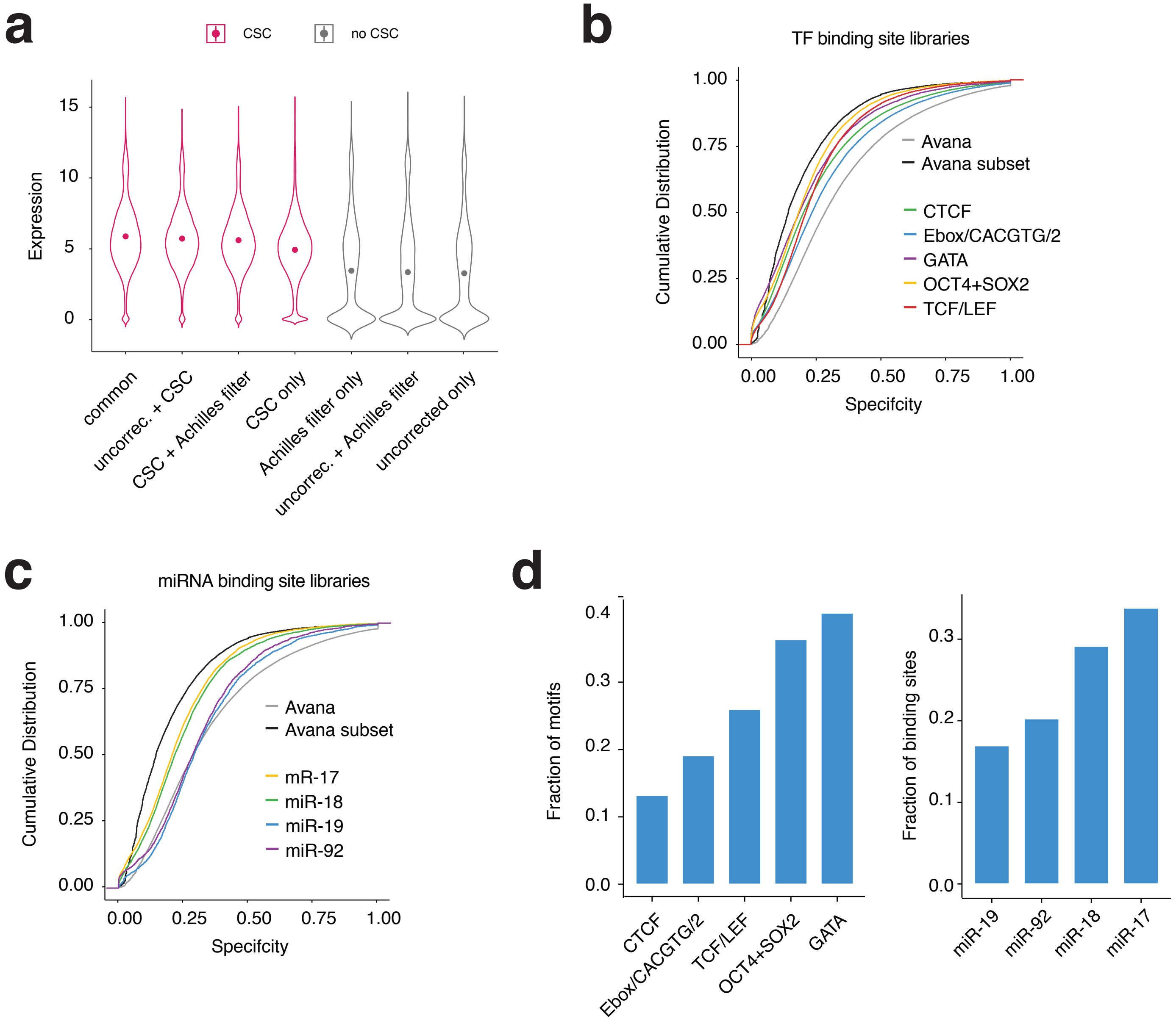

Perez Sup. Fig.5

a

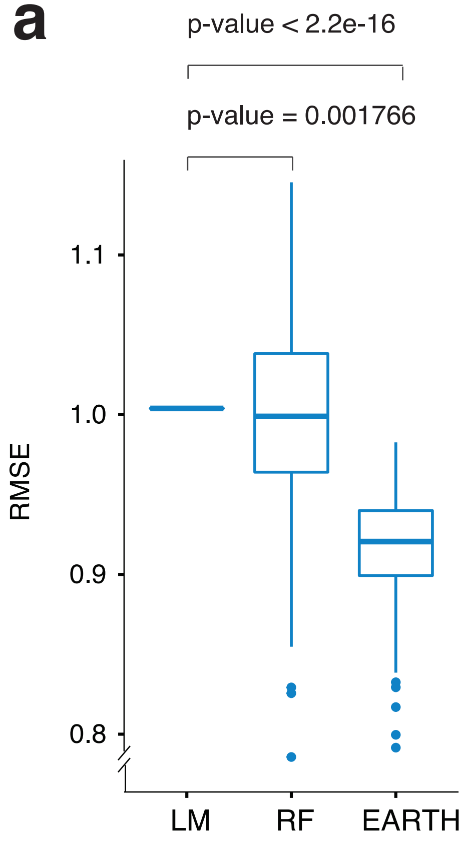

LM = Linear Lasso

RF = Random Forrest

EARTH = Multivariate Adaptive regression spline

Supplementary Figure Legends

Supplementary Figure 1. Unspecific gRNAs are preferentially depleted during CRISPR-Cas9 viability screens. (a) Cumulative Distribution of z-scores of log_2FC for gRNAs targeting known non-essential genes and binned based on their specificity (grey curves). The z-score distributions of gRNAs targeting known essential (red curve) and known non-essential genes (green curve) with high specificity (score=1) are also plotted as a reference. For gRNAs targeting non-essential genes, all curves differ significantly from that of specific gRNAs targeting non-essential genes except for gRNAs with a specificity equal or above 0.16 (blue curve; Kolmogorov–Smirnov test, adjusted for multiple testing). For simplicity, a single curve above this threshold is plotted corresponding to gRNAs with specificities between 0.16 and 0.17. **(b)** As in (a) but binning gRNAs based on the number of perfect target sites (H0) they have in the genome. **(c)** As in (a) but plotting only gRNAs with a single perfect target site in the genome (H0=1) and binned based on the number of target sites with a single mismatch. **(d)** As in (a) but binning gRNAs based on the Sum of their Hamming Neighbors. **(e-g)** Gene set enrichment analysis for the A375 melanoma cell line screen showing that genes targeted by promiscuous gRNAs (defined as specificity < 0.16 (e), specificity < 0.10 (f), or presence of multiple perfect targets (H0 > 1, (g)) are significantly enriched in high BF values. Each line of the plot represents a gene in the set, ordered by decreasing BF values. Gene sets were defined by number of promiscuous gRNAs targeting a gene. Only significant gene sets are shown. NES, normalized enrichment score. padj, p value adjusted for multiple testing. NES and padj for GSEA shown in e-g were calculated using the FGSEA R package against 100,000 random gene sets.

Supplementary Figure 2. Screen performance after off-target correction by CSC. (a) Area under the curve for precision-recall curves of all Project Achilles Avana screens (n= 689) before and after correction. **(b)** Example precision-recall curves for cell lines in which CSC improves (HS729) or decreases (LI7) the recall at 5%FDR. The AUC values for each curve are shown. **(c)** Boxplots showing precision, recall, and number of false positive hits in DepMap Avana screens (n= 689) when Bayes Factor (BF) values are varied to make the total number of genes identified as hits identical between the two pipelines. **(d)** Cumulative Distribution of median Distortion Ratio for all screens of the 19Q4 Project Achilles dataset. Curves show screens that have higher (magenta) or lower (green) recall at 5% FDR after CSC correction. **(e)** Boxplot showing recall values at 5% FDR for the entire 19Q4 dataset when data is uncorrected, corrected for off-targets with CSC, and simultaneously corrected for off-targets and copy number alteration by sequential use of CSC and CRISPRcleanR. Each dot represents the median recall value of a lineage (n= 26). All boxplots show minimum, maximum, median, first and third quartiles. p-values of boxplots p-values were calculated using a two-sided Wilcoxon test.

Supplementary Figure 3. CSC improves the performance of CRISPR-Cas9 essentiality screens. (a) Recall values at 5% FDR for each cell line in the 19Q4 dataset before (grey) or after correction (yellow). **(b)** Recall values before and after correction, plotted by lineage. Only lineages with more than 10 screens are shown.

Supplementary Figure 4. CSC performance over highly promiscuous libraries. (a) Violin plots showing the expression levels (log2(TPM+1)) of genes in the cell lines in which they were identified as dependencies. The common dataset shows genes identified by all three pipelines in a subset of cell lines. Dot represents the mean value. **(b)** Cumulative distributions of GuideScan specificity scores for all gRNAs cutting within transcription factor archetypal motifs overlapping consensus DNaseI footprints. The specificity distribution for Avana or the Avana gRNA subset used in Figure 3 are plotted as references. **(c)** As in (b) but for gRNA libraries design to cut within binding site motifs for miRNAs of the *miR-17~92* cluster. **(d)** Fraction of transcription factor

archetypal motifs (left) or miRNA binding sites (right) that become untargatable after filtering out unspecific gRNAs.

Supplementary Figure 5. Comparison of regression models on Project Achilles data. (a) RMSE values after regressing the mean depletion values of each gRNA against its 5 specificity metrics using three distinct regression models (Linear Lasso, Random Forrest, and EARTH). Boxplots show minimum, maximum, median, first and third quartiles RMSE values across entire Project Achilles 19Q4 dataset (n= 689). p-values were calculated using a two-sided Wilcoxon test.

Supplementary Notes

CSC software corrects off-target mediated gRNA depletion in CRISPR-Cas9 essentiality screens

Alexendar R. Perez[1,2], Laura Sala[1], Richard K. Perez[3,4], Joana A. Vidigal*[,1]

[1] Laboratory of Biochemistry and Molecular Biology, National Cancer Institute, Bethesda, MD, USA

[2] Department of Anesthesia and Perioperative Care, University of California, San Francisco, San Francisco, California, USA

[3] School of Medicine, University of California, San Francisco, San Francisco, California, USA

[4] Department of Anesthesiology, Perioperative and Pain Medicine, Stanford University, Palo Alto, California, UCA

Supplementary Note 1

The confounding effect of off-targeting has been well documented in CRISPR assays and, over the past several years, numerous research efforts have aimed at defining the tolerance of Cas9 to mismatches[15, 35, 36]. As a result, we now have a fairly comprehensive understanding of how the number, position, and type of mismatches interfere with both Cas9 binding[37] and endonucleolytic cleavage[15, 35-37], leading to the development of scores such as CFD[15] and Elevation[36] which describe how likely a potential off-target sequence is to be cleaved based on its similarity to the gRNA.

Despite this wealth of knowledge, the strategies employed to identify the genomic sequences that constitute potential off-target loci—on which CFD and Elevation scores are calculated—can be inaccurate[16]. Specifically, identification of potential sites of off-target cleavage is often achieved with alignment tools. However, tools such as Bowtie[26], Bowtie2[27], STAR[28], and BLAT[25] have a

trade-off between speed and exhaustive read-matching, often leading to the truncation of a search if an effort limit is exceeded[25-28]. This feature allows tools to process queries quickly, an essential criterion when working with large datasets. Yet, in the context of CRISPR it means that exhaustive identification of off-target sites is not always guaranteed particularly for highly promiscuous gRNAs[15]. Indeed, we have attempted to find potential off-targets for the most promiscuous gRNA in Avana with several alignment tools but were unable to identify a set of parameters for any of them that would accurately enumerate potential off-targets for this guide when using the sequence of the gRNA with an appended 'NGG' PAM at the 3' end as the query (see Methods). In Supplementary Data 1, we show a subset of the optimization tests we attempted along with the results they generated, including those used by BAGEL2 in their off-target search (see Methods). In contrast to previously reports[15], we were unable to find Bowtie2 parameters that returned all perfect matches nor all alignments with a single mismatch to the gRNA. In fact, we were only able to identify all perfect target sites using the BWA aligner. Although BWA did not return alignments with mismatches to our query using the sets of parameters described in Supplementary Data 1, we note that this is a sophisticated and well-established aligner that has been specifically developed to return fast and accurate alignments of short sequences, and therefore particularly suited to the off-target search task. We have not performed an exhaustive assessment of all BWA parameter settings and their outputs, and therefore it is likely that a specific combination not tested here would have retrieved accurate enumerations for mismatched off-targets. Given the behavior of BWA towards ambiguous bases, optimal off-target enumerations likely require users to use the gRNA sequence as their query as opposed to the target sequence as done in this study. This approach would require users to subsequently filter for alignment coordinates that are adjacent PAM motifs and that constitute potential off-targets for gRNA. Nevertheless, we would like to highlight that the vast parameter space that can be deployed for alignments and the requirement that users optimize the set of parameters to maximize alignment accuracy over speed may further

contribute to the vast discrepancies in the outputs of aligner-based gRNA off-target search tools commonly thought to perform comprehensive off-target searches[38]:

Supplementary Table 1: Comparison of off-target enumerations by commonly used tools for the most promiscuous gRNA in the Avana library:

Tool	Reference	Reported H0	Chromosome data	Algorithm
E-CRISP (evaluation mode)	39	241	ND	Bowtie2 aligner
CRISPko (CRISPick)	40	1,584*	ND	ND
CHOPCHOP	41	1,200	ND	Bowtie aligner
GuideScan	this work	288,646	no _alt	retrieval tree
GuideScan	16	301,892	all	retrieval tree
Cas-OFFinder (RGEN Tools)	42	288,148	no _alt, _random, or chrUn_	hash tree
BSgenome	43	131	chrUn_	
BSgenome	43	367	_random	
BSgenome	43	13,246	_alt	
BSgenome	43	288,148	no _alt, _random, or chrUn_	
BSgenome	43	301,892	all	

ND, not described; *, based on the number of on-target sequences identified. No additional off-targets reported; H0, number of perfect sites reported by each tool.

In contrast, both GuideScan[16] and Cas-OFFinder[42]—which do not utilize aligners in their search algorithm—accurately enumerate potential gRNA off-target loci, yielding identical counts to those of an exhaustive search performed with the BSgenome R package[43].

Project Achilles performs alignments using Bowtie as part of their off-target search method[2], and that likely accounts for the underestimation of gRNA promiscuity in the Avana library in the Achilles off-target filter (see also Supplementary Data 2).

Supplementary Note 2

The work from the Greenleaf, Kundaje, and Bassik labs previously defined a 0.2 GuideScan specificity cut-off to define unspecific gRNAs[9], instead of the 0.16 we define in this work. This discrepancy originates from the GuideScan tries used to enumerate gRNAs off-targets and consequently the specificity score of each gRNA. The original tries[16]—which were used by our colleagues in their work—were constructed using genome assemblies that contained alternative loci representations. This inadvertently over-estimated the number of off-targets for some gRNAs since the same locus could be represented by more than one coordinate in the assembly. To address this issue, we re-computed the tries for mouse and human genomes using the primary assemblies which exclude alternative coordinates and should represent a non-redundant haploid genome. We find that calculating the specificity threshold below which we see off-target mediated gRNA depletion leads to a value of 0.16 using these new tries but 0.2 when we use the original ones.
