# Supplementary material for: CSC software corrects off-target mediated gRNA depletion in CRISPR-Cas9 essentiality screens
Source: Nat Commun. 2021 Nov 9;12:6461. doi: 10.1038/s41467-021-26722-w (PMC8578331; doi:10.1038/s41467-021-26722-w)
Supplement: Supplementary file 2 — Description of Additional Supplementary Files [file 41467_2021_26722_MOESM2_ESM.pdf]

File Name: Supplementary Data 1

Description: Evaluation of alignment tools for gRNA off-target search

File Name: Supplementary Data 2

Description: Comparison of CSC and Project Achilles' off-target enumerations for Avana. First 2 columns contain gRNA descriptors, column 3-7 contain off-target information used by CSC, columns 8 and 9 contain off-target information described in the Achilles filter file.

File Name: Supplementary Data 3

Description: Off-target description for the Sanger library. First 2 columns contain gRNA descriptors, column 3-7 contain off-target information calculated by GuideScan.

File Name: Supplementary Data 4

Description: Example metrics file generated during the correction of an Avana screen

File Name: Supplementary Data 5

Description: Example metrics file generated during the correction of a Project Score screen
