# Supplementary material for: CSC software corrects off-target mediated gRNA depletion in CRISPR-Cas9 essentiality screens
Source: Nat Commun. 2021 Nov 9;12:6461. doi: 10.1038/s41467-021-26722-w (PMC8578331; doi:10.1038/s41467-021-26722-w)
Supplement: Supplementary file 8 — Reporting Summary [file 41467_2021_26722_MOESM8_ESM.pdf]

Corresponding author(s): Joana A Vidigal

Last updated by author(s): Sep 8, 2021

Reporting Summary

Nature Research wishes to improve the reproducibility of the work that we publish. This form provides structure for consistency and transparency in reporting. For further information on Nature Research policies, see our Editorial Policies and the Editorial Policy Checklist.

Statistics

For all statistical analyses, confirm that the following items are present in the figure legend, table legend, main text, or Methods section.

n/a	Confirmed	
☐	☒	The exact sample size (*n*) for each experimental group/condition, given as a discrete number and unit of measurement
☒	☐	A statement on whether measurements were taken from distinct samples or whether the same sample was measured repeatedly
☐	☒	The statistical test(s) used AND whether they are one- or two-sided<br>*Only common tests should be described solely by name; describe more complex techniques in the Methods section.*
☐	☒	A description of all covariates tested
☐	☒	A description of any assumptions or corrections, such as tests of normality and adjustment for multiple comparisons
☐	☒	A full description of the statistical parameters including central tendency (e.g. means) or other basic estimates (e.g. regression coefficient) AND variation (e.g. standard deviation) or associated estimates of uncertainty (e.g. confidence intervals)
☐	☒	For null hypothesis testing, the test statistic (e.g. *F*, *t*, *r*) with confidence intervals, effect sizes, degrees of freedom and *P* value noted<br>*Give P values as exact values whenever suitable.*
☒	☐	For Bayesian analysis, information on the choice of priors and Markov chain Monte Carlo settings
☒	☐	For hierarchical and complex designs, identification of the appropriate level for tests and full reporting of outcomes
☐	☒	Estimates of effect sizes (e.g. Cohen's *d*, Pearson's *r*), indicating how they were calculated

Our web collection on statistics for biologists contains articles on many of the points above.

Software and code

Policy information about availability of computer code

Data collection

no software was used for data collection.

Data analysis

Data was analyzed with the following software:

1. CSC Software and implementation.
The CSC was packaged in Python (version 2.7.15 & version 3.8.8) with Avana, Brunello, and GeckoV1, GeckoV2, Project Score, and DepMap libraries as package data. Pickle files for hg38 and mm10 genomes are also provided in a repository, to allow CSC to be implemented for any custom human or mouse libraries. We also provide a Docker image. All these files are freely available to download from our bitbucket repository (https://bitbucket.org/arp2012/csc_public/src/master/CSC_beta_python3/).

2.The Bagel software (version 0.91) was used to infer gene essentiality based on log2-fold changes of gRNAs for each gene.

3.To benchmark CSC, we compared its performance against the current approach of filtering out gRNAs suspected of off-target activity as implemented by Project Achilles. Information about this filter (which can be downloaded from the DepMap data repository as "Achilles_dropped_guides.csv") is provided here as part of Supplementary Table 3

4. To benchmark CSC off-target enumeration, we compared it with the output of aligners and CRISPR off-target identification approaches, include those used by BAGEL2. The parameters used in these searches are described in Supplementary Note 1 and Supplementary Table2.

5. GSEA analysis was performed using the the FGSEA R package (version 3.13)

6. Off-target enumeration built into the CSC software were calculated using the Guidescan software (version 1.0)

April 2020

7. Aligners tested in of target search: BLAT (version 3.5), Bowtie (version 1.3.0), Bowtie2 (version 2.4.4), BWA (version 0.7.17), STAR (version 2.7.9a)

8. Area Under the Curve (AUC) was calculated using the 'PRROC' R Package (version 1.3.1)

9. Copy number corrections were performed with CRISPRcleanR (version 1.0)

For manuscripts utilizing custom algorithms or software that are central to the research but not yet described in published literature, software must be made available to editors and reviewers. We strongly encourage code deposition in a community repository (e.g. GitHub). See the Nature Research guidelines for submitting code & software for further information.

Data

Policy information about availability of data

All manuscripts must include a data availability statement. This statement should provide the following information, where applicable:

- Accession codes, unique identifiers, or web links for publicly available datasets
- A list of figures that have associated raw data
- A description of any restrictions on data availability

Data Availability:
1. The human genome sequence used to enumerate potential off-target sites was downloaded from UCSC genome database (https://genome.ucsc.edu/)

2. The DepMap data used in this study is available at the DepMap project data repository (https://figshare.com/articles/DepMap_19Q4_Public/11384241/2) for screens performed with the Avana library, and at the Project Score page (https://score.depmap.sanger.ac.uk/downloads) for screens performed with the Sanger library.

3. RNA-seq TPM gene expression data (log2-transformed using a pseudo-count of 1) for protein coding genes can be downloaded from the DepMap project data repository (https://figshare.com/articles/DepMap_19Q4_Public/11384241/2).

4. Binding site predictions for miRNAs expressed by the miR-17~92 cluster can retrieved from TargetScan.
Bed files for transcription factor motif archetypes overlapping consensus DNaseI footprints can downloaded from (https://www.vierstra.org/resources/dgf).

Field-specific reporting

Please select the one below that is the best fit for your research. If you are not sure, read the appropriate sections before making your selection.

☒ Life sciences ☐ Behavioural & social sciences ☐ Ecological, evolutionary & environmental sciences

For a reference copy of the document with all sections, see nature.com/documents/nr-reporting-summary-flat.pdf

Life sciences study design

All studies must disclose on these points even when the disclosure is negative.

Sample size	No sample size calculation was performed. All screens in the DepMap 19Q4 and Project Score datasets were used in the analysis.
Data exclusions	No data was excluded from the analysis
Replication	This does not apply. All analysis was based on freely available data in the above mentioned databases.
Randomization	This does not apply. No samples/organisms/participants were allocated into experimental groups
Blinding	This does not apply. No group allocations were done.

Reporting for specific materials, systems and methods

We require information from authors about some types of materials, experimental systems and methods used in many studies. Here, indicate whether each material, system or method listed is relevant to your study. If you are not sure if a list item applies to your research, read the appropriate section before selecting a response.

Materials & experimental systems

n/a	Involved in the study
☒	☐ Antibodies
☒	☐ Eukaryotic cell lines
☒	☐ Palaeontology and archaeology
☒	☐ Animals and other organisms
☒	☐ Human research participants
☒	☐ Clinical data
☒	☐ Dual use research of concern

Methods

n/a	Involved in the study
☒	☐ ChIP-seq
☒	☐ Flow cytometry
☒	☐ MRI-based neuroimaging
